# Isolation and characterization of Shiga toxigenic *Escherichia coli* of animal and bird origin by multiplex polymerase chain reaction

**DOI:** 10.14202/vetworld.2016.123-127

**Published:** 2016-02-08

**Authors:** S. Neher, A. K. Hazarika, L. M. Barkalita, P. Borah, D. P. Bora, R. K. Sharma

**Affiliations:** 1 Department of Veterinary Microbiology, College of Veterinary Science, Assam Agricultural University, Guwahati, Assam, India; 2 Department of Animal Biotechnology, College of Veterinary Science, Assam Agricultural University, Guwahati, Assam, India

**Keywords:** *eae*, *Escherichia coli*, Shiga toxigenic *Escherichia coli*, Shiga toxin 1, Shiga toxin 2

## Abstract

**Aim:**

The purpose of this study was to determine the virulence genes and serotype of Shiga toxin producing *Escherichia coli* (STEC) strains isolated from animals and birds.

**Materials and Methods:**

A total of 226 different samples *viz*., fecal, intestinal content, rectal swab and heart blood were collected from different clinically affected/healthy animals and birds and were streaked on McConkeys’ lactose agar and eosin methylene blue agar for isolation of *E. coli*, confirmed by staining characteristics and biochemical tests. By polymerase chain reaction (PCR) all the *E. coli* isolates were screened for certain virulence genes, *viz*., Shiga toxin 1 (*stx*1), *stx*2 and *eae* and enterohemolytic (Ehly) phenotype was observed in washed sheep blood agar plate. All the isolated *E. coli* strains were forwarded to the National *Salmonella* and *Escherichia* Centre, Central Research Institute, Kasauli (Himachal Pradesh) for serotyping.

**Results:**

Out of 226 samples 138 yielded *E. coli*. All the isolates were screened for molecular detection of different virulent genes, *viz*. *stx1*, *stx*2 and *eae*, based on which 36 (26.08%) were identified as STEC. Among those STEC isolates, 15 (41.67%), 14 (38.89%), 1 (2.78%) exhibited *eae*, *stx2*, *stx1* alone, respectively, whereas 4 (11.11%) and 2 (5.56%) carried both *stx1* and *stx2*, *stx2* and *eae*, respectively. Among the STEC isolates 22 were belonged to 15 different sero-groups, *viz*., O2, O20, O22, O25, O43, O60, O69, O90, O91, O95, O106, O118, O130, O162 and O170 and others were untypable. Ehly phenotype was observed in 10 (27.78%) the STEC isolates.

**Conclusion:**

The present study concluded that STEC could be isolated from both clinically affected as well as healthy animals and birds. Regular monitoring of more samples from animal and bird origin is important to identify natural reservoir of STEC to prevent zoonotic infection.

## Introduction

Shiga toxigenic *Escherichia coli* (STEC) is considered to be the most common foodborne zoonotic pathogen causing various disease conditions in both animals and humans [1]. Ruminants are the most important source of STEC with cattle being regarded as the primary reservoir [2-4]. In humans, STEC infections may primarily result from consumption of undercooked beef, raw milk, meat and dairy products, vegetables, unpasteurized fruit juices, and water contaminated with feces of animal [5,6]. The virulent strains of STEC are associated with one or more types of Shiga toxin (stx1, stx2 or stx2 variants) as well as the property of producing intimin, which is required for attachment effacement lesions encoded by *eae* gene [2,7]. There are, at least, 200 serotypes of *E. coli* that are capable of producing shigatoxins [8-10]. However, of these serotypes *E. coli* O157:H7 is the most well-known to both microbiologists and the general public, but several non-O157 STEC strains are also associated with the production of shigatoxins [1,2,8,11-14].

Isolation of O157:H7, O157:H- or the other STEC serotypes from dairy cattle emphasize the role of raw milk as an important vehicle of transmission [3,15]. In animals, STEC is associated with hemorrhagic colitis (HC), hemolytic uremic syndrome (HUS), and edema disease in pig [1,16-18]. Pathogenic strains of *E. coli* that cause diseases are difficult to distinguish from those constitute a part of the normal intestinal flora of animals and man. Therefore, it is imperative to characterize the isolates in terms of their virulence factors so as to establish their pathogenic significance. Pathogenic strains of *E. coli* can be identified by detection of their toxins and toxin genes.

Considering the public health importance of STEC, it is necessary to isolate and characterize the STEC strains circulating in a particular region, so that appropriate control strategies could be adopted. This paper reports about characterization of STEC isolated from animals and birds of Assam, the Northeastern state of India based on molecular detection of certain virulence genes in the isolates.

## Materials and Methods

### Ethical approval

Ethical approval for the study was obtained from Institutional Animal ethics Committee of College of Veterinary Science, Assam Agricultural University, Khanapara.

### Source of sample

A total of 226 different samples, *viz*., fecal, intestinal content, rectal swab, and heart blood were collected aseptically in sample collection vial from apparently healthy and clinically ill/dead animals and birds from organized and some un-organized farms in and around Guwahati city.

### Isolation and identification of *E. coli*

All samples were primarily inoculated in McConkeys’ lactose agar plates and, later on, lactose fermenting colonies were subcultivated on eosin methylene blue agar medium to observe characteristic metallic sheen. Further identification was done on the basis of staining procedure and different biochemical tests as described by Edwards and Ewing [19].

### Serotyping of *E. coli*

The isolated *E. coli* strains were serotyped on the basis of somatic (O) and flagellar (H) antigens in the National *Salmonella* and *Escherichia* Centre, Central Research Institute, Kasauli (Himachal Pradesh) as per method of Edwards and Ewing [19].

### Molecular characterization by multiplex polymerase chain reaction (M-PCR)

All the *E. coli* isolates were screened for certain virulence genes, *viz*., *stx1*, *stx2* and *eae* genes by multiplex PCR [20]. The template DNA was obtained from each *E. coli* isolate by hot cold lysis procedure as per the method described by Titball *et al.*, 1989 [21]. Briefly, 16-18 h growth of *E. coli* were obtained in 2 ml Luria Bertoni broth. Broth cultures were centrifuged at 12,000 rpm for 10 min at 4°C. The pellets were resuspended in 75 μl of tris ethylene-diamine-tetraacetic acid buffer and heated in boiling water bath at 100°C for 20 min, followed by snap chilling in ice for 20 min and finally centrifuged at 12,000 rpm for 20 min at 4°C. The supernatant was directly used as template DNA for PCR for screening of virulence genes. The extracted DNA was subjected to multiplex PCR for screening of *stx*1, *stx*2 and *eae* genes using specific primers (Table-1). PCR was carried out with a 25.0 μl final reaction volume in ×2 Dream Taq Green PCR Master mix (Fermentas, USA), comprising 4 mM MgCl_2,_ 0.4 mM of each deoxynucleotides, 0.05 units/ml of *Taq* DNA polymerase, 150 mM tris-HCL PCR buffer, 0.5 μl (10 pmole/μl) each of primer for *stx*1 and *eae* gene and 1.0 μl (20 pmole/μl) of primer for *stx*2 gene. The thermal cyclic condition was 96°C for 4 min, 35 cycles of 95°C for 20 s, 57°C for 20 s, 72°C for 1 min followed by 72°C for 7 min in a gradient thermal cycler (Techne, Germany).

**Table-1:** Details of primers used for PCR reaction.

Primers	Sequence (5’-3’)	Target gene	Amplicon (bp)	References
*stx1* (F)	CAG TTA ATG TGG TGG CGA AGG	*stx1*	348	[22]
*stx1* (R)	CAC CAG ACA ATG TAA CCG CTG			
*stx2* (F)	ATC CTA TTC CCG GGA GTT TAC G	*stx2*	584	[22]
*stx2* (R)	GCG TCA TCG TAT ACA CAG GAG C			
*eae* (F)	TCA ATG CAG TTC CGT TAT CAG TT	*eae*	482	[20]
*eae* (R)	GTA AAG TCC GTT ACC CCA ACC TG			

PCR=Polymerase chain reaction, *stx*=Shiga toxin

The PCR amplified products were separated by electrophoresis at 85 V for 1 h with 1.8% (w/v) agarose gel containing ethidium bromide (0.5 μg/ml) in ×1 tris-borate-EDTA along with gene ruler 100 bp DNA ladder (Fermentas, USA) as a molecular weight marker and visualized as a single compact band of expected size under ultraviolet light in gel documentary system (Kodak, Germany).

### *In-vitro* expression of enterohemolysin (Ehly)

All the 36 (26.08%) STEC strains identified by PCR was tested for production of Ehly by inoculating on washed sheep blood agar plates supplemented with 10 mM CaCl_2_ as per Beutin *et al.*, 1989 [23]. After streaking, the plates were incubated at 37°C and examined at 4 and 24 h intervals. *E. coli*, which produced hemolytic reaction after 4 h and clear zone after 24 h, was considered as alpha-hemolytic, whereas strains producing small turbid hemolytic zone around the streaking line after 18-20 h were considered as Ehly. Non-hemolytic strains gave no reaction either after 4 h or 24 h.

## Results

**Figure-1:**
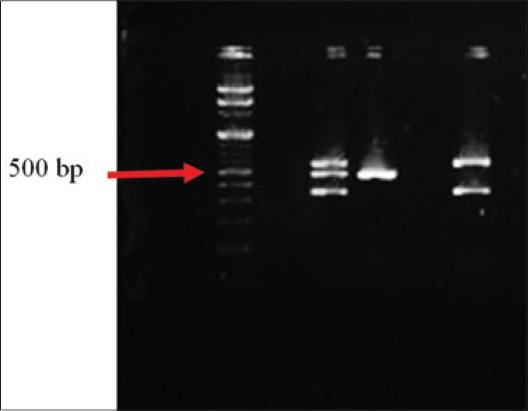
Gel electrophoresis picture of multiplex polymerase chain reaction of Shiga toxigenic *Escherichia coli*. Lane 1= 100 bp DNA ladder (Fermentus) Lane 2= Negative control (NTC) Lane 3= Positive control (548 bp, 482 bp, 348 bp) Lane 4= Test isolate positive for *eae* (482 bp) Lane 6= Test isolate positive for both *stx*1 and *stx*2 (348 bp, 584 bp)

A total of 226 different samples, *viz*., fecal, intestinal content, rectal swab, and heart blood were collected from apparently healthy and clinically ill/dead animals and birds. All the samples were tested for isolation of *E. coli*. Out of all the samples 138 (61.06%) were found to be positive for *E. coli* irrespective of the health status of the host. All the isolates were tested for the presence of certain virulence genes *viz. stx1*, *stx2* and *eae* by Multiplex-PCR (Figure-1). Among the 138 isolates a total of 36 (26.08%) isolates were identified as STEC. The highest isolation of STEC was exhibited by the clinically affected animals (19), followed by apparently healthy animals (12), clinically ill birds (3), and apparently healthy birds (2). Among the STEC isolates, 15 (41.67%) exhibited *eae* gene alone, 14 (38.89%) were positive for *stx*2 alone, both *stx*1 and *stx*2 were found in 4 (11.11%), 2 (5.56%) isolates exhibited *stx2* and *eae* together while 1(2.78%) isolate showed *stx1* alone (Table-2). Among all the 36 STEC isolates 22 belonged to 15 different serogroups, *viz*., O2, O20, O22, O25, O43,O60, O69, O90, O91, O95, O106, O118, O130, O162 and O170 and others were either untypable or rough strain irrespective of type and health status of hosts. On *in-vitro* Ehly expression study, 17 (47.22%) out of 36 STEC isolates exhibited the Ehly phenotype (*E-Hly*) on washed sheep blood agar supplemented with CaCl_2_. All the 17 isolates they were found to be belonging to the serogroups O2, O22, O60, O69, O90, O91, O95 and O118with *stx*1, *stx*2 or *eae* gene singly or in combination.

**Table-2:** Isolation and molecular characterization of STEC of animal and bird origin.

Source	Health status	Number of samples screened	Number of samples positive for *E. coli*	Number of *E. coli* isolates	Number of STEC isolates	Number of isolates positive for virulence gene				

						*stx1*	*stx2*	*stx1*+*stx2*	*stx2+eae*	*eae*
Ani-mal	Clinically ill/dead	84	58 (42.03)	58	19	-	14	4	2	11
	Apparently healthy	85	50 (36.23)	50	12					
Bird	Clinically ill/dead	32	14 (10.14)	14	3	1	-	-	-	4
	Apparently healthy	25	16 (11.59)	16	2					
Total		226	138 (61.06)	138	36 (26.08)	1 (2.78)	14 (38.89)	4 (11.11)	2 (5.56)	15 (41.67)

Figures in the parenthesis indicate percentages. STEC=Shiga toxigenic *Escherichia coli*, *stx*: Shiga toxin

## Discussion

STEC an emerging human pathogen of public health concern, are able to cause serious disease in humans including mild non-bloody or severe bloody diarrhea, HC, life-threatening condition HUS and thrombocytopenic purpura; in pig it is associated with edema disease. Transmission of STEC to humans can occur as a result of direct contact with STEC-contaminated fecal material, from handling or petting animals, or by exposure to focally contaminated mud or vegetation during recreational activities. Pathogenic strains of *E. coli* can be identified by detection of their toxins and toxin genes. *E. coli* O157:H7 serotype is the most well-known STEC serotype but several other non-O157:H7 is also important in terms of virulence. A detection of different genes of STEC in adult cattle, calves, sheep, pigs and goats by PCR was also reported by previous workers [24,25]. The *stx*1, *stx*2 and *eae* genes encode for Stx1, Stx2 and intimin respectively, which are the characteristics cytotoxins released by STEC. In the present study, *eae* gene was found in the highest numbers followed by *stx2* gene. A high prevalence of *eae* positivity reflects the presence of locus for enterocyte attaching and effacing lesions, responsible for host colonization and virulence by STEC [26]. Strains carrying *eae* and *stx2* are considered to be more pathogenic to humans and especially *stx2* is being the most important virulence factor associated with the human disease like HUS and HC [8,27]. Ehly production is also associated with STEC infection, and those strains are called as Ehly *E. coli*. It has been suggested that *stx*, *eae* gene and Ehly might have synergistic action in causing disease during STEC infection [28]. The present findings showed a close association between *stx* and E-hlyproduction. These findings assumed a special significance in terms of potential public health hazards as these may be cross transferred to man via food chain.

## Conclusion

The present study was undertaken to isolate and characterize STEC from animals and birds for zoonotic significance. The serotypes found in the present study might be of zoonotic importance. STEC could be isolated from both clinically affected as well as healthy animals and birds. The highest numbers of STEC could be isolated from clinically affected animals and birds than healthy individual. Multiplex PCR used in this study provides a simple, highly sensitive, specific and rapid technique for detection of STEC-mediated disease. The epidemiological and zoonotic significance of STEC isolates need special emphasis for improved diagnosis, control and surveillance measures.

## Authors’ Contributions

AKH and PB designed the experiment. SN and LMB collected sample and performed the experiment. Manuscript preparation was supervised, reviewed and edited by RKS and DPB. All authors read and approved the final manuscript.
